# Efficient Ammonium Nitrogen Metabolization and γ-PGA Production by *Bacillus velezensis* GY1 Isolated from Swine Manure Digestate

**DOI:** 10.3390/microorganisms14040729

**Published:** 2026-03-24

**Authors:** Hong-Ping Chen, Jia-Zhou Li, Jin-Yan Li, Zhi-Lin Wang, Jun-Jin Deng, Xue-Ming Dan

**Affiliations:** 1 Guangdong Laboratory for Ling Nan Modern Agriculture, College of Marine Sciences, South China Agricultural University, Guangzhou 510642, China; chplyn14@163.com; 2 Key Laboratory of Animal Nutrition and Feed Science in South China, Ministry of Agriculture and Rural Affairs, Guangdong Provincial Key Laboratory of Animal Breeding and Nutrition, Guangdong Engineering Technology Research Center of Animal Meat Quality and Safety Control and Evaluation, Institute of Animal Science, Guangdong Academy of Agricultural Sciences, Guangzhou 510640, China; lijiazhou@gdaas.cn (J.-Z.L.); jinyan__li@163.com (J.-Y.L.); 3 Guangdong Laboratory for Lingnan Modern Agriculture Heyuan Sub-Center, Heyuan 517500, China; 4 Agro-Biological Gene Research Center, State Key Laboratory of Swine and Poultry Breeding Industry, Guangdong Academy of Agriculture Sciences, No. 20 Jinying Road, Tianhe, Guangzhou 510640, China; wangzhilin@gdaas.cn

**Keywords:** *Bacillus velezensis* GY1, ammonium nitrogen removal, γ-PGA synthesis, nitrogen upcycling

## Abstract

Efficient microbial assimilation of high-concentration ammonium nitrogen and its conversion into value-added bioproducts represent a pivotal yet underexplored strategy for sustainable nitrogen management. Here, we report a newly isolated *Bacillus velezensis* strain, GY1, with a robust intrinsic capacity for simultaneous NH_4_^+^-N assimilation and γ-polyglutamic acid (γ-PGA) biosynthesis. Under optimized conditions (37 °C, pH 7.0, C/N = 12:1), GY1 achieved 76.5% removal of ammonium nitrogen (400 mg/L) with negligible nitrite accumulation (<0.02 mg/L), indicating assimilation rather than nitrification. Transcriptomic analysis revealed a coordinated metabolic flux wherein the glutamine synthetase - glutamate synthase pathway GS-GOGAT pathway supplies glutamate for γ-PGA synthesis, while polymerization further facilitates ammonium sequestration via electrostatic interactions. GY1 produced up to 612.8 mg/L γ-PGA, and genetic overexpression of *capB* synchronized these pathways, enhancing both ammonium assimilation (87.4%) and γ-PGA yield (843.9 mg/L). Notably, this metabolic coupling remained resilient in complex substrates, achieving 68.8% ammonium removal and 220.7 mg/L γ-PGA production in untreated biogas slurry. Together, these findings establish GY1 as a metabolically robust platform linking nitrogen assimilation with biopolymer synthesis, offering a mechanistic framework for circular nitrogen economies.

## 1. Introduction

The unprecedented expansion of intensive livestock and poultry farming worldwide has triggered a massive discharge of nitrogenous wastewater, posing a profound threat to global biogeochemical cycles. Ammonium nitrogen (NH_4_^+^-N) pollution, in particular, is among the most serious and escalating challenges in environmental governance. Over the past three decades, anthropogenic ammonium nitrogen emissions have exhibited a persistent upward trajectory, posing threats to ecosystem integrity and climate stability [1]. Excessive nitrogen accumulation can induce widespread harmful algal blooms that release potent toxins, posing substantial threats to human health and ecological stability [2]. Moreover, wastewater containing certain nitrogen compounds can seep into aquifers, exacerbating the problem and polluting groundwater resources [3,4]. Traditional biological nitrogen removal (BNR) processes rely on a continuous biochemical process of aerobic nitrification (NH_4_^+^ → NO_2_^−^ → NO_3_^−^), which is primarily completed by slow-growing autotrophic bacteria (usually taking more than 15 h), followed by anaerobic denitrification (NO_3_^−^ → NO_2_^−^ → NO → N_2_O → N_2_), which requires a large amount of organic carbon. Although this theory is scientifically sound, its practical implementation remains challenging [5]. In particular, conventional BNR has several limitations when used to treat high-ammonium nitrogen wastewater, including high energy demand, reliance on external carbon sources, the risk of nitrite accumulation, irreversible conversion of nitrogen into nonrecoverable N_2_, and the inability to achieve nitrogen resource recovery [6].

In recent decades, research regarding ammonium nitrogen pollution treatment has shifted from simple nitrogen removal to nitrogen resource recovery and value-added utilization. Physicochemical strategies, such as the precipitation of magnesium ammonium nitrogen phosphate (struvite, MgNH_4_PO_4_·6H_2_O), offer a pathway to generate slow-release fertilizers, although they are often hindered by the high cost of magnesium reagents [7,8]. In addition to physicochemical recovery strategies, biological approaches have been explored [9]. Several studies have demonstrated that near-complete purification of high-strength ammonium nitrogen wastewater (100 mg L^−1^) can be achieved by constructing a microalgal–bacterial symbiotic system, with ammonium nitrogen removal efficiency reaching up to 99.5% [10]. The effectiveness of this system relies on the establishment of an internal material cycle, in which microalgal photosynthesis supplies dissolved oxygen for bacterial nitrification, while bacterial metabolism provides inorganic carbon (CO_2_) and essential nutrients to support microalgal growth [11]. The alkali- and salt-tolerant yeast strain *Debaryomyces hansenii* JL8-0 can efficiently produce single-cell protein (SCP) under conditions of pH 8.5 and 2500 mg L^−1^ NH_4_^+^-N, thereby achieving efficient nitrogen recovery [12]. Although these approaches demonstrate the feasibility of nitrogen recovery to a certain extent, they are often constrained by stringent environmental requirements, high economic costs, limited economic value, and complex process configurations. Alternative strategies that integrate pollutant removal with value-added product generation have attracted increasing interest [13,14].

γ-Polyglutamic acid (γ-PGA) is a biodegradable, nontoxic, and water-soluble biopolymer with broad application potential in agriculture, food, and medical fields [15,16,17,18] but is associated with high production costs as its industrial production relies predominantly on pure cultures and defined substrates (e.g., glutamate). Fermentative production of γ-PGA using nitrogen sources in wastewater (e.g., ammonium salts) represents an attractive strategy to simultaneously reduce raw material expenses and wastewater treatment costs, aligning well with the principles of a circular economy. *Bacillus subtilis* NX-2 can efficiently assimilate ammonium nitrogen from wastewater via anabolic pathways for γ-PGA synthesis. Following adaptive evolution, this strain exhibited ammonium nitrogen and nitrite removal efficiencies of up to 98.03% and 93.62%, respectively [19]. In addition, metabolically engineered strains, such as *Bacillus amyloliquefaciens*, have been shown to utilize nonfood carbon sources (e.g., crude glycerol) in combination with ammonium nitrogen to achieve efficient γ-PGA production, with titres reaching 60.4 g L^−1^ [20]. Collectively, these findings highlight the feasibility of microbial conversion of ammonium nitrogen in wastewater into high value-added γ-PGA, offering a promising strategy that integrates environmental remediation with resource recovery.

Despite these preliminary successes, few strains have been reported to efficiently assimilate ammonium nitrogen from wastewater and directly convert it into γ-PGA. The underlying molecular mechanism remains unclear, and the feasibility and performance of their application in actual high-ammonium nitrogen wastewater (complex composition and low C/N ratios) need to be verified. In this study, we isolated a novel strain, *Bacillus velezensis* GY1 (GDMCC No: 64792), from livestock wastewater. It exhibits outstanding efficiency in removing high concentrations of ammonium nitrogen while concurrently demonstrating a robust ability to synthesize γ-PGA using ammonium ions. The core objectives of this study are threefold: (1) to systematically optimize the environmental and nutritional parameters (e.g., temperature, pH, C/N ratio, and initial ammonium nitrogen concentration) governing the ammonium nitrogen removal efficiency of strain GY1; (2) to elucidate the biochemical pathway responsible for ammonium nitrogen transformation and γ-PGA synthesis in GY1, employing a combination of genomic and transcriptomic analyses, followed by experimental validation of the proposed route; and (3) to rigorously evaluate the translational potential of strain GY1 by assessing its performance in terms of ammonium nitrogen removal and γ-PGA production using real, complex anaerobic digestion effluent (biogas slurry) as the primary growth substrate. This study aims to elucidate the high-strength ammonium assimilation mechanism of *B*. *velezensis* GY1 and its efficient conversion into value-added γ-PGA, providing a biological basis for future high-nitrogen wastewater resource recovery.

## 2. Materials and Methods

### 2.1. Media

Bacterial strain screening was conducted using two distinct media formulations: the heterotrophic nitrification medium (HNM) and the aerobic denitrification medium (ADM). Heterotrophic nitrification medium (g/L): MgSO_4_·7H_2_O (3.0 g), K_2_HPO_4_ (4.8 g), NaCl (2.0 g), (NH_4_) _2_SO_4_ (0.5 g), MnSO_4_·4H_2_O (0.05 g), sodium succinate (3.0 g), FeSO_4_ (0.05 g), and pH adjusted to 7.5. Aerobic denitrification medium (g/L):KH_2_PO_4_ (1.0 g), sodium succinate (3.0 g), KNO_3_ (1.2 g), MgSO_4_·7H_2_O (1.5 g), agar (15 g), and pH adjusted to 7.5. The basal M9 medium contained (g/L) Na_2_HPO_4_ (12.8 g), KH_2_PO_4_ (3.0 g), NaCl (0.5 g), an NH_4_Cl (1.0 g) supplemented with 1 M CaCl_2_ (10 μL), 1 M MgSO_4_ (0.2 mL), and a trace element solution (25 μL) containing (g/L) FeCl_3_·6H_2_O (0.27 g), ZnCl_2_·4H_2_O (0.02 g), CoCl_2_·6H_2_O (0.02 g), Na_2_MoO_4_·2H_2_O (0.02 g), CaCl_2_·2H_2_O (0.01 g), CuCl_2_·6H_2_O (0.013 g), H_3_BO_3_ (0.005 g), and concentrated HCl (1 mL). Carbon and nitrogen sources were subsequently supplemented as required for specific experimental conditions.

The biogas slurry used for microbial cultivation was collected from a pig farm storage tank at the Heyuan Branch Center, Lingnan Modern Agriculture Guangdong Laboratory. The raw slurry was characterized by an alkaline pH of 7.8 and a high ammonium nitrogen (NH_4_^+^-N) concentration of 479.4 mg/L. To prepare the medium, the crude slurry was first centrifuged at 10,000× *g* for 10 min at 4 °C to eliminate suspended solids and particulate impurities. The resulting supernatant underwent sequential sterilization via vacuum filtration through 0.45 μm and 0.22 μm pore-size membranes (e.g., Millipore, Billerica, MA, USA). This clarified, sterile filtrate was subsequently utilized as the base medium for assessing the NH_4_^+^-N removal performance and metabolic stability of *B. velezensis* GY1.

### 2.2. Microbial Isolation

Wastewater samples (specifically swine manure digestate) were collected from a swine-farming facility at the Institute of Animal Sciences, Tianhe District, Guangzhou, for the isolation of ammonium nitrogen-removing bacteria. The target bacterial strain was isolated from the wastewater sample by subjecting it to three successive rounds of purification on heterotrophic nitrification (HNM) and aerobic denitrification (ADM) media, following the method described by Li et al. [21]. Finally, the isolates were cultured in LB broth overnight and cryopreserved in 15% glycerol (*v*/*v*) at −80 °C.

### 2.3. Microbial Identification

Following cultivation of strain GY1 to the logarithmic growth phase, cells were harvested by centrifugation (5000× *g*, 10 min) and resuspended in phosphate buffer. After serial dilution, samples were fixed with 2.5% (*v*/*v*) glutaraldehyde at 4 °C for 24 h prior to microscopic morphological characterization using standardized protocols. Genomic DNA extraction was performed with the OMEGA Bacterial DNA Kit (Omega Bio-Tek, Norcross, GA, USA), followed by high-throughput sequencing conducted by Sangon Biotech (Shanghai, China) Co., Ltd. The raw genome sequences were subjected to Average Nucleotide Identity (ANI) analysis via the EzBioCloud ANI Calculator platform, with species identification determined through whole-genome alignment coverage (>95% identity threshold) against the Type Strain Genome Server (TYGS) database.

### 2.4. Univariate Optimization of the Ammonium Nitrogen Removal Performance of Strain GY1

To optimize the ammonium nitrogen removal efficiency of strain GY1, a single-factor experimental design was employed to evaluate the effects of carbon source (glucose, lactose, sucrose, sodium acetate, and disodium succinate), carbon-to-nitrogen (C/N) ratio (ranging from 4 to 20 at intervals of 4 with a fixed nitrogen concentration of 400 mg/L), and pH (6.0, 7.0, and 8.0). Bacterial suspensions were inoculated at 2% (*v*/*v*) into 250 mL Erlenmeyer flasks. The C/N ratio was adjusted by varying the carbon concentration, and the pH was adjusted using HCl or NaOH solutions. All treatments were conducted in triplicate, and ammonium nitrogen removal efficiency was determined spectrophotometrically at 625 nm.

### 2.5. The Detection of Ammonium Nitrogen Ions and Nitrites

Ammonium nitrogen ion concentrations were determined using the indophenol blue colorimetric assay. After centrifugation of 48 h GY1 cultures at 12,000× *g* for 10 min, the supernatants were diluted with deionized water to within the linear range of the assay. Aliquots (20 μL) were transferred to 96-well microplates containing 100 μL of Reagent A (phenol–ethanol solution, 1:1, *v*/*v*) and 100 μL of Reagent B (alkaline sodium hypochlorite solution containing 0.15 M NaOH, pH 12.5). Following incubation at 25 °C for 1 h, 20 μL of Reagent C (nitroprusside solution, 0.05%, *w*/*v*) was added, and absorbance was measured at 625 nm using a microplate reader. Ammonium nitrogen concentrations were calculated based on an ammonium nitrogen sulfate standard curve (y = 0.2374x − 0.067, R^2^ = 0.9971) over the range of 0–10 μg/mL NH_4_^+^-N. The ammonium nitrogen removal efficiency was calculated as follows: ammonium nitrogen removal efficiency (%) = (C_0_ − C_t_)/C_0_ × 100% (where C_0_ is the initial ammonium nitrogen concentration and C_t_; is the final ammonium nitrogen concentration). The nitrogen conversion rate (NCR) was calculated according to the following equation: NCR (%) = C_γ-PGA_ × 0.1085/∆C_NH4_^+^_-N_ × 100%. C_γ-PGA_ (mg/L) is the concentration of produced γ-PGA, and ∆C_NH4_^+^_-N_ (mg/L) represents the mass concentration of the consumed ammonium nitrogen (the difference between the initial and residual NH_4_^+^-N). The constant 0.1085 represents the theoretical nitrogen mass fraction in the γ-PGA monomer (C_5_H_7_NO_3_).

We employed the diazotization method for nitrite quantification: pH-adjusted samples (60 μL) were mixed with 70 μL of a sulfanilic acid solution (1% in 3 M HCl) and 70 μL of N-(1-naphthyl) ethylenediamine dihydrochloride (0.1% *w*/*v*), incubated for 15 min at 25 °C, then measured at 540 nm against sodium nitrite calibration standards with a calibration curve of y = 0.4037x − 0.0937, with R^2^ = 0.9947 (0–5 μg/mL NO_2_^−^-N).

### 2.6. Transcriptome Sample Preparation and Analysis

For RNA-seq sample preparation, the experimental and control groups utilized ammonium nitrogen sulfate and ammonium nitrate as nitrogen sources, respectively. When cell growth reached an OD600 of 1, cells were harvested by centrifugation at 8000 rpm for 10 min. Bacterial RNA was immediately extracted following the E.Z.N.A.^®^ Bacterial RNA Kit protocol (Omega Bio-Tek, Norcross, GA, USA). RNA concentration and integrity were assessed using a Fragment Analyzer, with three biological replicates per condition. For RNA sequencing, the RNA samples were delivered to Wuhan BGI Tech Solutions Co., Ltd. (Wuhan, China) for high-throughput sequencing on the DNBSEQ platform. After quality control filtering of raw sequences obtained from RNA-seq, alignment was performed against the genome of *B*. *velezensis* GY1, achieving a coverage > 90%. Genes showing significant differential expression between the experimental and control groups were selected based on a fold change threshold of |log_2_FC| > 1 and *p* < 0.05. Transcriptome sequences were compared against sequences in the KEGG database using the Blast tool (https://blast.ncbi.nlm.nih.gov/Blast.cgi, accessed 10 January 2026) to obtain single-gene KO numbers. KO numbers of upregulated and downregulated gene sets were mapped to the KEGG database of *B. velezensis*, obtaining pathway IDs. The top 10 pathways significantly enriched in the distribution of upregulated and downregulated genes were ranked based on gene counts per pathway.

### 2.7. Detection of γ-Polyglutamic Acid

The molecular weight of γ-PGA was analyzed using a Waters 2695 HPLC system (Waters Corp., Milford, MA, USA) with a TSK gel 2000SWxl column (300 mm × 7.8 mm). The mobile phase comprised acetonitrile, water, and trifluoroacetic acid (40/60/0.1, *v*/*v*) at a flow rate of 0.5 mL/min and a column temperature of 30 °C. A standard curve was constructed using reference standards: thyroglobulin bovine, γ-globulins from bovine blood, albumin chicken egg grade VI, and ribonuclease A type I-A (all from Sigma-Aldrich, St. Louis, MO, USA). The calculation formula was Log (molecular weight [n]) = 8.76 − 0.208 Rt (retention time).

The γ-PGA content was determined by cetyltrimethylammonium bromide (CTAB) spectrophotometry, which relies on the formation of an insoluble complex between the cationic surfactant CTAB and negatively charged polyglutamic acid, followed by absorbance measurement at 400 nm [22]. GY1 fermentation broth cultured for 48 h was centrifuged at 8000 rpm for 15 min to remove cells. Ten millilitres of supernatant was mixed with three volumes of absolute ethanol, precipitated at 4 °C for 18 h, and centrifuged at 8000 rpm for 15 min. The pellet was dissolved in distilled water, diluted according to the standard curve range, mixed with an equal volume of 0.07 M CTAB in a 2% NaOH solution, and incubated for 3 min, and turbidity was measured at 400 nm using a spectrophotometer.

### 2.8. FTIR Detection and Analysis

Fourier transform infrared spectroscopy (FTIR) was employed to characterize the GY1 culture supernatant and identify their functional groups and chemical structural features. The sample was thoroughly ground with dried potassium bromide (KBr) at a mass ratio of approximately 1:100 and then compressed into a transparent pellet under hydraulic pressure. The spectrum was recorded in transmittance mode over a wavenumber range of 4000 to 400 cm^−1^ with a resolution of 4 cm^−1^, accumulating 32 scans per measurement to enhance the signal-to-noise ratio.

### 2.9. Statistical Analysis

All experimental assays were performed in at least three independent biological replicates. Data are expressed as the mean ± standard deviation (SD). Statistical differences between treatment groups were determined using one-way analysis of variance (ANOVA), followed by Tukey’s post hoc test for multiple comparisons. Statistical significance was predefined at a *p*-value < 0.05. Data processing and visualization were executed using Microsoft Excel and GraphPad Prism 9.0 (GraphPad Software, San Diego, CA, USA). Additionally, metabolic network diagrams and conceptual schematics were refined and illustrated using Adobe Illustrator 2021.

## 3. Results

### 3.1. Isolation and Genomic Characterization of B. velezensis GY1 as a High-Efficiency Ammonium Nitrogen Removal Strain

**Figure 1:**
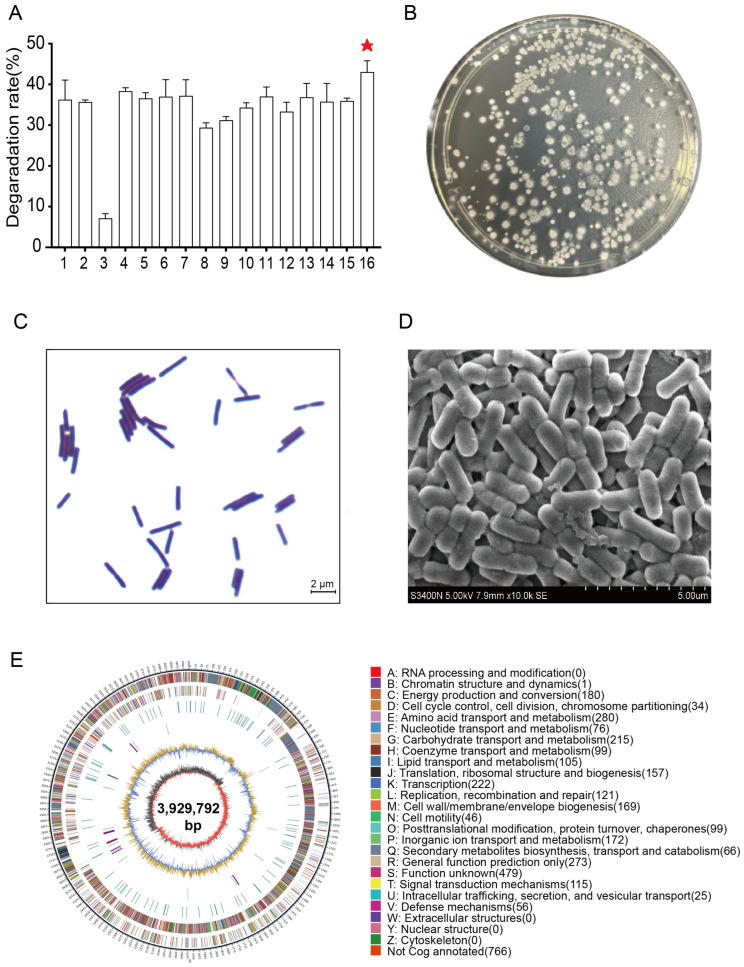
Isolation and identification of *B. velezensis* GY1 with functional screening. (**A**) Comparative ammonium nitrogen removal efficiency of *Bacillus* spp., the red asterisk indicates the strain with the highest nitrogen removal efficiency; (**B**) colony morphology of GY1 on LB agar; (**C**) Gram-positive staining of GY1 (1000×); (**D**) scanning electron micrograph of GY1 cells; (**E**) circular genomic map of GY1.

A total of 16 *Bacillus* strains were selectively enriched and isolated from livestock and poultry wastewater, among which strain GY1 exhibited the greatest NH_4_^+^-N removal rate (43.05%) over the other strains (7.1–38.3%) (Figure 1A). Morphological analysis revealed that GY1 is a Gram-positive, short, rod-shaped bacterium that forms creamy-white, opaque colonies on LB agar (Figure 1B,C). Scanning electron microscopy unveiled an average cell length of 2.22 ± 0.16 μm and a width of 0.77 ± 0.02 μm (Figure 1D), which are dimensions consistent with those of typical *Bacillus* species [23].

Furthermore, whole-genome sequencing resolved the taxonomic identity of GY1, revealing a circular chromosome of 3,929,792 bp with a GC content of 46.5% (Figure 1E). The 16S rDNA sequence of GY1 showed 100% BLASTn (https://blast.ncbi.nlm.nih.gov/Blast.cgi, accessed 10 January 2026) identity against that of the NCBI reference strains, confirming its designation as *B*. *velezensis*. Functional annotation (RAST/KEGG) revealed key genes involved in nitrogen metabolism, including assimilatory nitrate reductase (*nasA*), glutamine synthetase (*glnA*), glutamate dehydrogenase (*gudB*), and γ-polyglutamate synthetase (*capBCA*), along with dissimilatory reductases (*narGHI* and *nirBD*). However, no hallmark genes for autotrophic nitrification (*amoA* and *hao*) or complete denitrification (*nor* and *nos*) were detected (Figure S1). This genetic profile suggests that GY1 primarily employs noncanonical ammonium nitrogen assimilation via the GS-GOGAT pathway, channelling NH_4_^+^ into glutamate synthesis and extracellular γ-PGA biopolymer production, rather than nitrification–denitrification processes.

### 3.2. Growth Optimization and Ammonium Nitrogen Removal Performance of B. velezensis GY1

**Figure 2:**
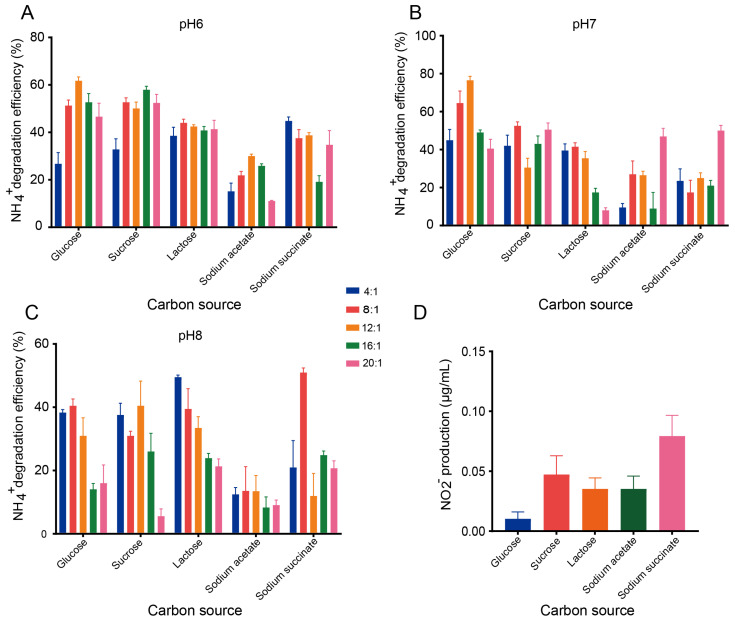
Effects of carbon source and C/N ratio on ammonium nitrogen removal efficiency and nitrite accumulation by *B. velezensis* GY1. (**A**–**C**) NH_4_^+^-N removal efficiency of strain GY1 under different carbon sources at pH 6 (**A**), pH 7 (**B**), and pH 8 (**C**) with C/N ratios of 4:1, 8:1, 12:1, 16:1, and 20:1. (**D**) Nitrite (NO_2_^−^) production by strain GY1 under the optimal ammonium nitrogen removal conditions for each carbon source. Error bars represent standard deviations of triplicate experiments.

The bacterial growth rate and NH_4_^+^-N removal efficiency are strongly influenced by key operational parameters, including the C/N ratio, pH and the carbon source [24]. The ammonium nitrogen removal efficiency of GY1 was systematically evaluated under various operational parameters (carbon source, pH, and C/N ratio). The ammonium nitrogen removal performance of strain GY1 varied with pH. Overall, at a pH of 6.0, relatively stable ammonium nitrogen removal efficiencies were observed across different carbon sources, with minimal differences among C/N ratios. With the exception of sodium acetate, most treatments achieved removal efficiencies of approximately 40%, with the highest reaching 61.8%. Notably, lactose exhibited comparable ammonium nitrogen removal efficiencies across all the tested C/N ratios, remaining at approximately 40% (Figure 2A). At a pH of 7.0, compared with the other carbon sources, glucose resulted in higher ammonium nitrogen removal efficiency, particularly at a C/N ratio of 12:1, where the removal efficiency reached 76.5%. Sucrose and lactose showed moderate effects, whereas sodium acetate and sodium succinate resulted in appreciable ammonium nitrogen removal only at a high C/N ratio (20:1) (Figure 2B). At a pH of 8.0, improved ammonium nitrogen removal was observed at C/N ratios of 4:1 and 8:1. Specifically, lactose at a C/N ratio of 4:1 and sodium succinate at 8:1 achieved removal efficiencies of approximately 50% (Figure 2C). Overall, higher C/N ratios resulted in increased ammonium nitrogen removal at pH values of 6.0 and 7.0, whereas at a pH of 8.0, relatively low C/N ratios also supported efficient ammonium nitrogen removal. In addition, glucose resulted in the highest ammonium nitrogen removal performance among the tested carbon sources. At pH values of 6.0 and 7.0, the ammonium nitrogen removal efficiency with glucose at C/N ratios of 8:1 and 12:1 exceeded 50%.

Nitrite production was quantified under the optimal ammonium nitrogen removal conditions for different carbon sources. Nitrite accumulation was low for all the tested carbon sources (Figure 2D). The highest nitrite concentration was observed with sodium succinate (0.076 µg/mL), whereas the lowest was detected with glucose (0.015 µg/mL). The concentrations of nitrite with sucrose, lactose, and sodium acetate did not exceed 0.05 µg/mL. On the basis of these results, glucose at a C/N ratio of 12:1 and a pH of 7 was selected as the optimal cultivation condition for subsequent experiments with strain GY1.

### 3.3. Transcriptomic Characterization of Ammonium Nitrogen Assimilation Pathways in B. velezensis GY1

**Figure 3:**
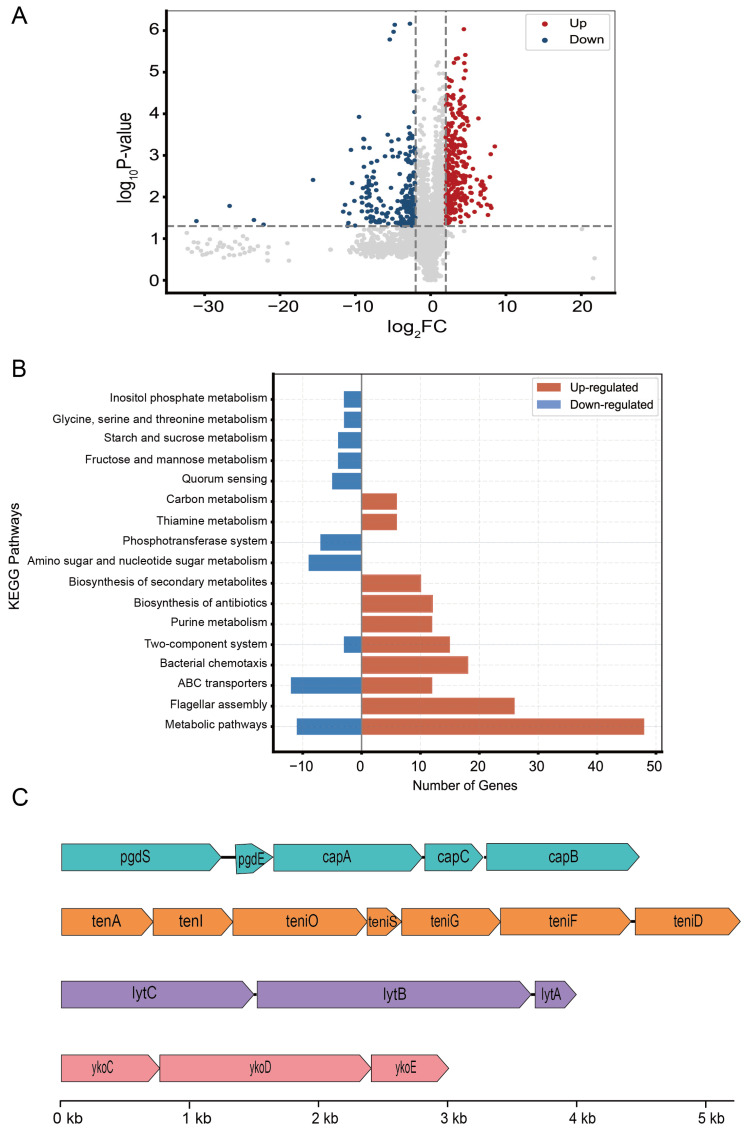
Transcriptomic profiling and functional analysis of *B. velezensis* GY1. (**A**) Volcano plot of differentially expressed genes (DEGs). Red and blue dots represent significantly up- and down- regulated genes, respectively (|log_2_FC| > 1 and *P_adj_* < 0.05), while grey dots indicate genes with no significant difference; (**B**) KEGG pathway enrichment analysis of DEGs; (**C**) gene cluster of the top 30 upregulated DEGs.

The transcriptomic analysis of GY1 focused on cellular growth and nitrogen metabolism. We conducted RNA sequencing of GY1 cultured with ammonium nitrogen sulfate or ammonium nitrate as the sole nitrogen source to explore transcriptional responses. Transcriptomic profiling of GY1 revealed 499 differentially expressed genes (DEGs), 290 of which were upregulated and 219 of which were downregulated (Figure 3A). KEGG enrichment analysis revealed that seventeen pathways were enriched with both upregulated and downregulated DEGs, most of which were primarily associated with major metabolic categories, such as metabolic pathways (map01100), purine metabolism, aminoglycan and nucleotide sugar metabolism, thiamine metabolism, and carbon metabolism (Figure 3B). These shared pathways suggest the formation of a functionally coordinated module.

Through further annotation of the upregulated DEGs in the KEGG-enriched pathways, we identified gene clusters directly or indirectly related to ammonium nitrogen assimilation. These include genes responsible for the synthesis of γ-PGA (*capBCA*, *pgdS*, and *pgdE*); genes involved in thiamine (vitamin B1) cofactor biosynthesis (*tenA*, *thiG*, *thiF*, and *thiC*); the transporter protein *ykoCDE*, which participates in central carbon metabolism and facilitates thiamine uptake; and *lytABC*, which participates in bacterial “programmed cell lysis” (Figure 3C). The synchronized induction of these genes resulted in the formation of clusters, supporting a mechanistic basis for ammonium nitrogen assimilation by GY1 under high-ammonium nitrogen conditions.

### 3.4. Metabolic Integration of γ-PGA Synthesis and NH_4_^+^-N Removal in B. velezensis GY1

**Figure 4:**
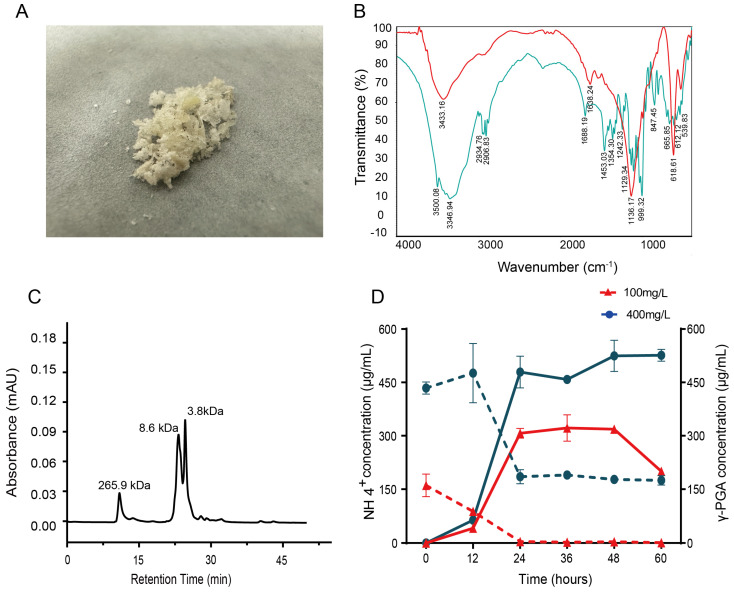
Metabolic flux linkage between γ-PGA synthesis and NH_4_^+^-N removal in *B*. *velezensis* GY1. (**A**) Crude extract of γ-polyglutamic acid obtained from the culture supernatant of GY1; (**B**) FT-IR analysis of γ-PGA produced by the wild-type strain (red) and the standard sample (blue); (**C**) molecular weight distribution of γ-PGA synthesized by GY1; (**D**) comparative NH_4_^+^-N removal capacity and γ-PGA yield of GY1 at 100 mg/L and 400 mg/L NH_4_^+^-N. The dashed line represents the NH_4_^+^ removal curve, and the solid line represents theγ-PGA production curve.

The transcriptome analysis revealed that free ammonium is assimilated into glutamine via glutamine synthetase and then converted to glutamate by glutamate synthase. Glutamate subsequently serves as the primary substrate for γ-PGA biosynthesis and is polymerized into chains that sequester excess nitrogen extracellularly. To verify the presence of γ-PGA, we performed crude extraction of γ-PGA from the denitrification-treated samples and characterized its structure using infrared spectroscopy (Figure 4A,B). The infrared spectra shown in Figure 4B compare the crude extract of the experimental group (red line) with the standard γ-PGA sample (blue line). Key absorption peaks corresponding to characteristic functional groups in γ-PGA are observed in both spectra, with notable peaks at 3436.94 cm^−1^ and 2934.76 cm^−1^ attributed to N-H and C-H stretching vibrations, respectively. The peak around 1681.19 cm^−1^ is indicative of the amide bond, whereas peaks at 1129.34 cm^−1^ and 999.32 cm^−1^ suggest the presence of C-O stretching. The overall profiles are similar, confirming the presence of γ-PGA in the crude extract. Slight differences in intensity and peak position may indicate variations in sample composition or the degree of purity.

Liquid chromatography–mass spectrometry (LC-MS) analysis revealed that GY1 predominantly synthesized low-molecular-weight (LMW) γ-PGA (<10 kDa), with major molecular weight peaks at 3.8 and 8.6 kDa, while a minor high-molecular-weight fraction (~265.9 kDa) was also detected (Figure 4C). This is atypical; strains normally produce high-molecular-weight (10–10,000 kDa) γ-PGA [19]. The dominance of LMW γ-PGA aligns with adaptive advantages under high-ammonium nitrogen conditions: reduced polymer size enhances solubility and permeability, facilitating rapid nitrogen efflux and minimizing intracellular accumulation. Further analysis indicated that the ammonium nitrogen removal efficiency of GY1 was positively correlated with the γ-PGA yield under different nitrogen loads (Figure 4D). At 160 mg/L NH_4_^+^-N, the γ-PGA yield was 199.8 mg/L (21.68% nitrogen conversion rate), and at 430 mg/L NH_4_^+^-N, the γ-PGA yield increased to 526.3 mg/L (22.05% nitrogen conversion rate), after which it eventually plateaued.

### 3.5. Transformation of NH_4_^+^-N in Digestate Wastewater by B. velezensis GY1

**Figure 5:**
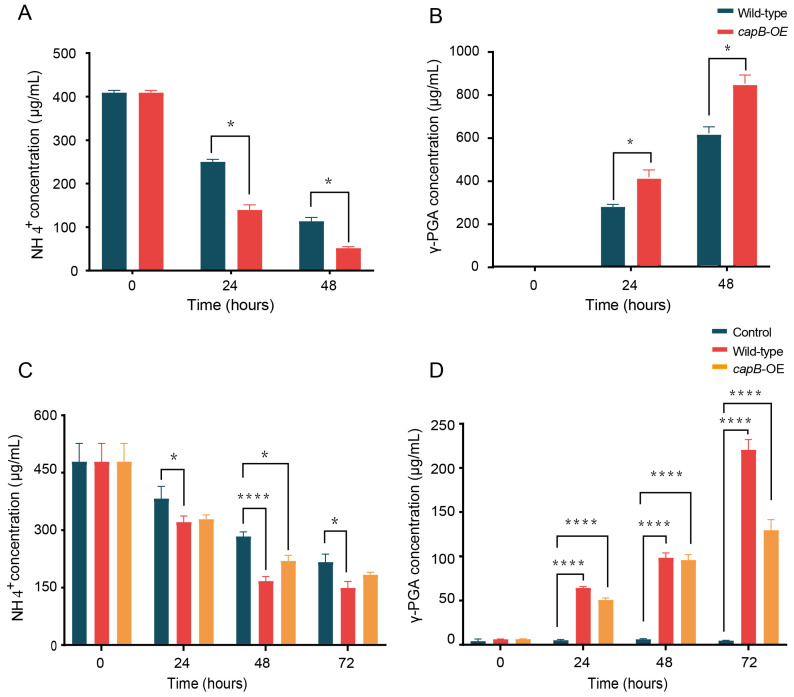
NH_4_^+^-N removal and γ-PGA production analysis of wild-type and *capB*-OE *B*. *velezensis* GY1 strains, (* *p* < 0.05, **** *p* < 0.0001). (**A**) The NH_4_^+^-N removal capacity of wild-type GY1 and the *capB*-overexpressing strain; (**B**) the γ-PGA production ability of wild-type GY1 and the engineered strain; (**C**) NH_4_^+^-N removal capacity of wild-type GY1 and the *capB*-overexpressing strain (pHY300-*pHpaII*-*capB*) in biogas slurry; (**D**) corresponding γ-PGA production under identical conditions in biogas slurry.

To further increase γ-PGA production and NH_4_^+^-N assimilation, a recombinant plasmid (pHY300PLK-*capB*) targeting the core synthase gene *capB* (also denoted as *pgsB*) was constructed in strain GY1. Given that the full-length *capBCA* gene is 2813 bp and encodes a large protein, its complete overexpression could impose a significant metabolic burden on the host bacteria. Previous studies have indicated that the isolated *pgsB* gene (a homologue of *capB*) plays a crucial role in γ-PGA synthesis and that overexpressing this gene alone can effectively increase production [25]. The ammonium nitrogen removal efficiencies of the recombinant strain (OD600 = 1.41) and the wild-type strain (OD600 = 2.17) were compared after 48 h of cultivation at an initial ammonium nitrogen concentration of 400 mg/L. Under these conditions, the performance of the recombinant strain significantly increased: ammonium nitrogen removal efficiency increased from 72.2% to 87.4%, while γ-PGA production increased by 27.4% (from 612.8 to 843.9 mg/L) (Figure 5A,B). These results indicate that γ-PGA synthesis directly drives ammonium nitrogen assimilation by channelling inorganic nitrogen into polymer-bound glutamate residues and that the engineered strain enhances the cell’s ammonium nitrogen assimilation capacity, thereby optimizing the carbon–nitrogen stoichiometric balance through metabolic reconfiguration.

*B*. *velezensis* GY1 exhibited high ammonium nitrogen removal efficiency in digestate wastewater, achieving an NH_4_^+^-N removal rate of 68.8% within 72 h (from 479.4 to 149.6 mg/L) without exogenous carbon supplementation (Figure 5C). The process was based on its dual metabolic strategy: ammonium ions are both assimilated into the cell for biosynthesis and used for the synthesis of γ-PGA (yield 220.7 mg/L), which is fixed extracellularly in a polymer form. Notably, *B. velezensis* GY1 demonstrates robust application potential for the efficient remediation and resource recovery of high-strength ammonium nitrogen in authentic digestate wastewater. The wild-type strain exhibited superior ammonium nitrogen removal efficiency and γ-PGA yield compared with the *capB* overexpression mutant, with a 30% higher removal rate and a 41.2% greater γ-PGA yield (220.7 mg/L vs. 129.8 mg/L, respectively) (Figure 5D). These results may be attributed to the complex composition and harsh environmental conditions of biogas slurry, in which cells must simultaneously cope with multiple survival challenges, including detoxification, osmotic stress regulation, and competition for nutrients. The diversion of cellular energy towards excessive product synthesis in engineered strains may compromise basal metabolism and stress tolerance, thereby reducing overall environmental adaptability.

## 4. Discussion

High-ammonium nitrogen environments have profound regulatory effects on bacterial denitrification efficiency through multiple mechanisms, including the inhibition of key functional enzymes, the reconfiguration of metabolic networks, and the restructuring of microbial community composition. Notably, GY1 achieved a removal efficiency of 76.5% even at an initial NH_4_^+^-N concentration of 400 mg/L, significantly exceeding the tolerance thresholds reported for many other bacteria, which often experience reduced specific activity and detrimental nitrite accumulation exceeding approximately 400 mg/L NH_4_^+^-N [26]. This is a notable finding given that GY1 reached the stationary phase within 24 h under these demanding conditions without significant nitrite accumulation. This mitigates a common failure point in BNR systems where nitrite accumulates when high ammonium nitrogen concentrations typically disrupt bacterial metabolism, induce oxidative stress, impair energy generation, and critically imbalance the expression of key genes involved in nitrification (e.g., *amoA*, *hao*, and *nxrA*) [27]. The resilience exhibited by GY1 implies the operation of robust assimilatory nitrogen metabolism, effectively avoiding these common inhibitory pathways. This metabolic robustness is likely supported by the coordinated action of the GS-GOGAT pathway coupled with γ-PGA synthesis, which together function as a primary nitrogen sink, channelling inorganic ammonium nitrogen into macromolecular storage, thereby mitigating the oxidative stress and energy disruption typically induced by NH_4_^+^-N oxidation or accumulation under high ammonium nitrogen loads.

Genus-specific enzymatic systems and the susceptibility of nitrogen transformation pathways to modulation by environmental conditions result in significant variations in ammonium nitrogen metabolism and removal efficiency, even at comparable initial ammonium nitrogen concentrations. For instance, at moderate initial NH_4_^+^-N concentrations of approximately 100 mg/L, *Pseudomonas* spp. consistently demonstrated near-complete (100%) removal within 24 h, while the removal efficiency of other genera ranged from 85% to 97.6%. *Bacillus* strains generally perform optimally under relatively low concentrations of NH_4_^+^-N (Table 1). By contrast, GY1 demonstrates exceptional versatility, exhibiting high performance across both low- and high-ammonium nitrogen regimes. At 100 mg/L NH_4_^+^-N, complete removal was achieved within 24 h, matching the efficacy of the prototypical denitrifying genus *Pseudomonas*.

**Figure 6:**
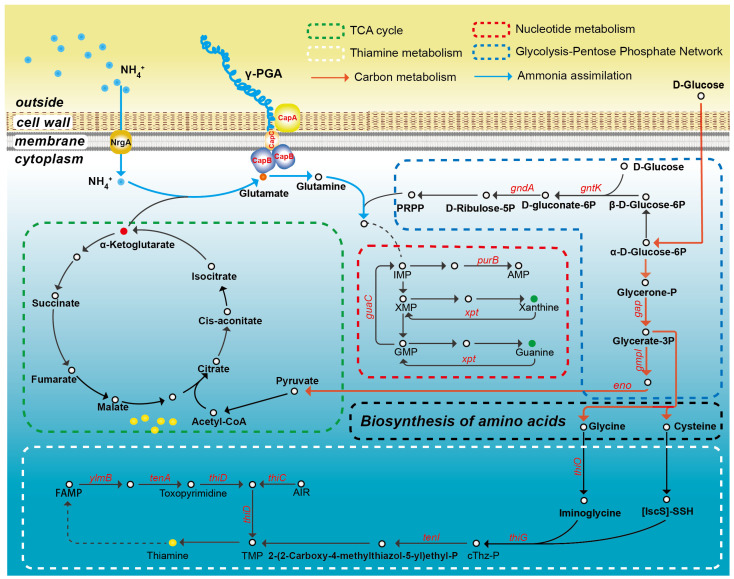
Metabolic pathway of γ-PGA synthesis in *B. velezensis* GY1 under excess nitrogen conditions, with a schematic representation of the genes involved in glycolysis, the tricarboxylic acid cycle, the pentose phosphate pathway, amino acid biosynthesis, and thiamine metabolism activated following ammonium intake.

Microbial ammonium nitrogen metabolism typically exhibits mechanistic diversity, encompassing both autotrophic pathways and heterotrophic assimilation. Such metabolic flexibility endows GY1 with enhanced adaptability in terms of nitrogen utilization, thereby enabling it to thrive under diverse environmental conditions [36,37]. GY1 demonstrates remarkable metabolic efficiency, with its architecture highlighting the synergy between central carbon metabolism and nitrogen assimilation (Figure 6). After glucose enters the cell, part of it undergoes glycolysis to form pyruvate, which enters the TCA cycle to generate α-ketoglutarate, while the other part is directed via 3-phosphoglycerate for amino acid biosynthesis, resulting in the production of glycine and cysteine. These amino acids are involved in serine metabolism and contribute to thiamine synthesis, thereby maintaining the function of the TCA cycle. In nitrogen assimilation, intracellular NH_4_^+^ combines with α-ketoglutarate from the TCA cycle under the catalysis of glutamate dehydrogenase or glutamate synthase to form glutamate. Glutamate can further react with NH_4_^+^ via glutamine synthetase to form glutamine. Both glutamate and glutamine serve as precursors for γ-PGA synthesis (catalyzed by the *capBCA* complex) and as substrates for nucleotide and protein biosynthesis. This coordinated metabolic network fulfils cellular biosynthetic demands while preventing the accumulation of toxic ammonium.

Under high-ammonium nitrogen stress, GY1 channels excess ammonium nitrogen to γ-PGA synthesis, a strategy that helps mitigate toxicity and supports cellular adaptation to environmental fluctuations [38]. The γ-PGA yield and ammonium nitrogen removal efficiency of strain GY1 increased concurrently with increasing nitrogen load. When the NH_4_^+^-N concentration increased from 160 mg/L to 430 mg/L, the γ-PGA yield increased from 199.8 mg/L to 526.3 mg/L, reaching equilibrium once the system stabilized. Analogous mechanisms have been observed in *Alcaligenes faecalis* JQ135, in which the Dirammox pathway (via DnfC-mediated GS) channels ammonium nitrogen into Glu/Gln precursors for γ-PGA synthesis [39]. Similarly, *Pichia kudriavzevii* HJ2 converts NH_4_^+^-N to LMW γ-PGA as a nitrogen-sequestration strategy [40]. The LMW γ-PGA produced by GY1 has significant biotechnological potential. Its low viscosity and high solubility enable applications that are inaccessible to its high-MW counterparts, such as nanoparticle drug delivery (enhanced tissue penetration) and nutrient-absorption boosters in agriculture [41,42]. In wastewater treatment, the dual function of GY1, 76.5% ammonium nitrogen removal at 400 mg/L NH_4_^+^-N coupled with γ-PGA synthesis, represents a potential circular bioeconomic approach. By transforming inorganic pollutants into biodegradable polymers, this process aligns with the focus on waste-to-resource innovation.

The inherent metabolic architecture of *B. velezensis* GY1, particularly its reinforced NH_4_^+^ → glutamate → γ-PGA biosynthetic pathway, positions it as a promising synthetic biological chassis for nitrogen valorization, enabling dual environmental objectives: mitigating aquatic eutrophication through efficient high-ammonium nitrogen detoxification and producing biodegradable γ-PGA with broad environmental applications. As a cationic chelator, γ-PGA effectively binds heavy metals (e.g., Cu^2+^ and Pb^2+^) in contaminated water, while its pronounced hygroscopic properties significantly increase soil moisture retention—a critical benefit in drought-prone agricultural regions [43]. The robustness and versatility of this platform are further evidenced by its compatibility with low-cost, complex feedstocks such as biogas slurry. This byproduct of livestock manure anaerobic digestion is rich in nitrogen, phosphorus, potassium, and micronutrients and may serve as a sustainable fertilizer [44]. However, untreated slurry poses environmental risks because of high ammonium nitrogen levels, residual antibiotics, heavy metals, and phosphates [45]. Importantly, compared with conventional γ-PGA production processes reliant on defined media, GY1 can utilize raw biogas slurry wastewater efficiently for direct γ-PGA synthesis without the need for external nitrogen supplementation.

While this study confirms the feasibility of using raw biogas slurry for γ-PGA production by GY1, the yields obtained under the initial, unoptimized conditions indicate the scope for improvement. This suboptimal productivity, compared with performance in defined or supplemented media, likely stems from the inherent complexity and variability of raw biogas slurry, which impose substrate limitations and suboptimal environmental conditions (e.g., potential C/N imbalance, unadjusted pH, variable temperature, and suboptimal aeration) that were not systematically addressed in this preliminary study. Future research will specifically target these limitations by applying previously optimized parameters for GY1 growth and γ-PGA synthesis (e.g., optimal temperature, pH, preferred carbon sources, and ideal C:N ratio) to fermentation strategies using biogas slurry as the primary medium. This targeted optimization approach, guided by fundamental metabolic understanding, is expected to significantly increase γ-PGA productivity. Subsequently, successful laboratory-scale processes will be scaled up to assess the technical feasibility and economic viability of nonsterile γ-PGA production from biogas slurry using GY1. This progression represents an important translational step towards realizing the industrial potential of this innovative waste-to-resource strategy.

## 5. Conclusions

This study isolated a strain of *B. velezensis*, GY1, and elucidated the efficacy and mechanism of GY1 in transforming high-ammonium nitrogen waste streams into value-added γ-PGA. The native regulatory network of GY1 enables efficient NH_4_^+^ assimilation via the GS-GOGAT cycle to glutamate, coupled with γ-PGA polymerization (*capBCA* genes). This pathway minimizes intracellular ammonium toxicity while achieving 68.8% NH_4_^+^-N removal in biogas slurry. GY1 converts waste ammonium nitrogen into low-molecular-weight γ-PGA (<10 kDa), which exhibits superior solubility for farming applications such as organic fertilizer production. This aligns with bioeconomy principles by valorizing waste nitrogen, which is an advance given that agricultural effluents account for 70% of global freshwater withdrawals.

## Figures and Tables

**Table 1:** Summary of highly efficient ammonium nitrogen-removing strains.

Strain	Source	Nitrogen Removal Efficiency	Reference
*B. velezensis* GY1	Wastewater	100 mg/L, 24 h, 100%	/
*Pseudomonas fluorescens* 2P24	Wheat fields	100 mg/L, 48 h, 97.64%	[28]
*Pseudomonas stutzeri* YG-24	Sediment	100 mg/L, 24 h, 100%	[29]
*Vibrio diabolicus* SF16	Sediment	120 mg/L, 42 h, 91.82%	[30]
*Rhodococcus* sp. CPZ24	Swine wastewater	100 mg/L, 25 h, 86%	[31]
*Enterobacter cloacae* DK-6	Pig farm	105.56 mg/L, 48 h, 86.98%	[32]
*Klebsiella pneumoniae* CF_S9	Domestic wastewater	120 mg/L, 24 h, 85%	[33]
*B.subtilis* H1	Mariculture ponds	42 mg/L, 82.39%	[34]
*B.simplex* Hb	Frozen soil	60 mg/L, 82.16%	[35]
*B.sp* N31	Shrimp ponds	20.01 mg/L, 42 h, 86.3%	[27]

## Data Availability

The original contributions presented in this study are included in the article/Supplementary Materials. Further inquiries can be directed to the corresponding authors.

## Supplementary Materials

The following supporting information can be downloaded at: https://www.mdpi.com/article/10.3390/microorganisms14040729/s1, Figure S1: Nitrogen metabolism pathway of *B. velezensis* GY1. The numbers in the boxes represent the EC numbers of key enzymes in the metabolic pathway, and the blue boxes highlight the coding genes corresponding to the related enzymes present in the genome.
