# Supplementary material for: Efficient Ammonium Nitrogen Metabolization and γ-PGA Production by Bacillus velezensis GY1 Isolated from Swine Manure Digestate
Source: Microorganisms. 2026 Mar 24;14(4):729. doi: 10.3390/microorganisms14040729 (PMC13118326; doi:10.3390/microorganisms14040729)
Supplement: Supplementary file 1 [file microorganisms-14-00729-s001.zip › microorganisms-4210693-supplementary.pdf]

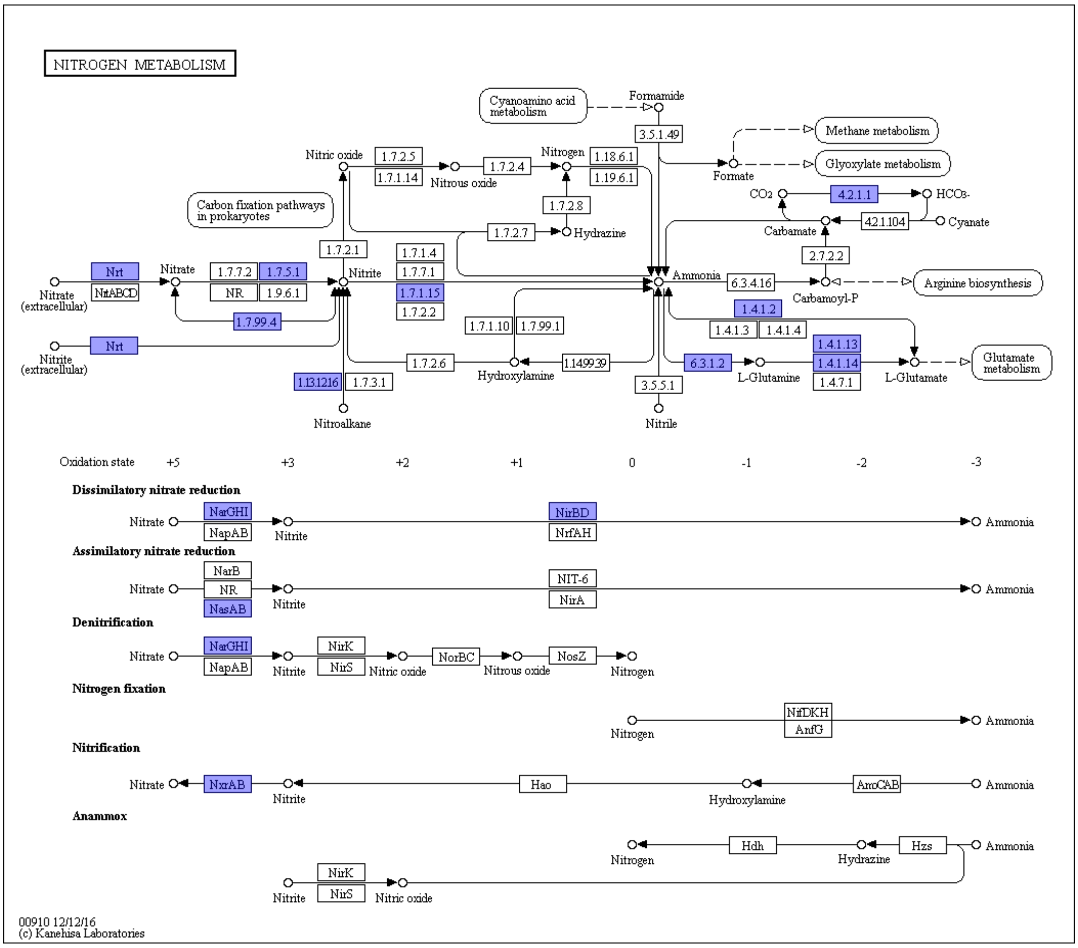

Figure S1. Nitrogen metabolism pathway of *B. velezensis* GY1. The numbers in the boxes represent the EC numbers of key enzymes in the metabolic pathway, and the blue boxes highlight the coding genes corresponding to the related enzymes present in the genome.
